# Muscle, functional and cognitive adaptations after flywheel resistance training in stroke patients: a pilot randomized controlled trial

**DOI:** 10.1186/s12984-016-0144-7

**Published:** 2016-04-06

**Authors:** Rodrigo Fernandez-Gonzalo, Sol Fernandez-Gonzalo, Marc Turon, Cristina Prieto, Per A. Tesch, Maria del Carmen García-Carreira

**Affiliations:** Department of Physiology and Pharmacology, Karolinska Institutet, Stockholm, Sweden; Research Department, Parc Taulí Hospital Universitari. Institut d’Investigació i Innovació Parc Taulí I3PT. Universitat Autònoma de Barcelona, Sabadell, Spain; Centro de Investigación Biomédica En Red de Enfermedades Respiratorias (CIBERES), Instituto de Salud Carlos III, Madrid, Spain; Department of Radiology, Parc Taulí Hospital Universitari. Institut d’Investigació i Innovació Parc Taulí I3PT. Universitat Autònoma de Barcelona, Sabadell, Spain; Diagnostic Imaging, Althaia Xarxa Assistencial Universitària de Manresa, Manresa, Spain; Department of Neurology, Parc Taulí Hospital Universitari. Institut d’Investigació i Innovació Parc Taulí I3PT. Universitat Autònoma de Barcelona, Sabadell, Spain

**Keywords:** Executive function, Eccentric overload, Muscle-brain interaction, Muscle hypertrophy

## Abstract

**Background:**

Resistance exercise (RE) improves neuromuscular function and physical performance after stroke. Yet, the effects of RE emphasizing eccentric (ECC; lengthening) actions on muscle hypertrophy and cognitive function in stroke patients are currently unknown. Thus, this study explored the effects of ECC-overload RE training on skeletal muscle size and function, and cognitive performance in individuals with stroke.

**Methods:**

Thirty-two individuals with chronic stroke (≥6 months post-stroke) were randomly assigned into a training group (TG; *n* = 16) performing ECC-overload flywheel RE of the more-affected lower limb (12 weeks, 2 times/week; 4 sets of 7 maximal closed-chain knee extensions; <2 min of contractile activity per session) or a control group (CG; *n* = 16), maintaining daily routines. Before and after the intervention, quadriceps femoris volume, maximal force and power for each leg were assessed, and functional and dual task performance, and cognitive functions were measured.

**Results:**

Quadriceps femoris volume of the more-affected leg increased by 9.4 % in TG. Muscle power of the more-affected, trained (48.2 %), and the less-affected, untrained limb (28.1 %) increased after training. TG showed enhanced balance (8.9 %), gait performance (10.6 %), dual-task performance, executive functions (working memory, verbal fluency tasks), attention, and speed of information processing. CG showed no changes.

**Conclusion:**

ECC-overload flywheel resistance exercise comprising 4 min of contractile activity per week offers a powerful aid to regain muscle mass and function, and functional performance in individuals with stroke. While the current intervention improved cognitive functions, the cause-effect relationship, if any, with the concomitant neuromuscular adaptations remains to be explored.

**Trial registration:**

Clinical Trials NCT02120846

## Background

Skeletal muscle is a leading target of secondary injury after stroke [1]. While causes explaining muscle deterioration are not completely understood, the sedentary lifestyle typically taken on by stroke survivors may add to the ameliorated lower limb muscle health caused by the injury *per se* [2]. To combat debilitating effects of stroke, resistance exercise (RE), favoring high-intensity muscle actions has shown efficacy [3]. Interestingly, individuals with stroke show markedly less reduction in eccentric (ECC; lengthening) than concentric (CON; shortening) muscle force [4]. Thus, while traditional RE presents an insufficient stimulus during the ECC action to optimize muscle adaptations in patients with stroke [4, 5], RE calling for maximal ECC actions boosts efficacy of training [6, 7]. Therefore, offering ECC overload during RE appears critical to promote the desired adaptations following stroke.

Flywheel RE was originally designed to maintain muscle health of astronauts during spaceflight [8]. It employs iso-inertial technology rather than gravity dependent weights, which allows for maximal CON and ECC muscle actions, with brief episodes of ECC overload [9]. Due to the energy storage characteristics of the inertial system, and by means of specific instructions to the trainee, the peak force generated during the ECC phase of the movement may be 15-30 % greater than what is produced in the preceding CON action [10]. Flywheel RE produces greater muscle hypertrophy and peripheral neural adaptations than weight-loaded RE in healthy subjects [9, 11]. Recently, we also showed that flywheel RE improves neuromuscular functions and physical abilities, without exacerbating spasticity in stroke victims [12]. This would suggest ECC-overload RE could serve as a highly effective rehabilitation tool following stroke.

In addition to functional and muscle alterations, up to 80 % of individuals with stroke show cognitive dysfunction [13]. This impedes vital daily-life activities, interferes with functional recovery, and hence increases dependency [13, 14]. Aerobic exercise training improves cognitive abilities in both older adults [15] and stroke survivors [16, 17]. In older adults, this effect appears amplified if aerobic exercise is combined with RE [15]. These results suggest RE *per se* facilitates activity of different cognitive domains. Indeed, aging individuals showed improved memory, executive functions, attention and conflict resolution after RE training [18, 19].

Due to different neural strategies, RE performed at variable velocity induces more profound adaptations (e.g. greater force gains) than constant velocity (i.e. isokinetic) muscle actions [20]. Compared with CON or isometric actions, executing ECC muscle actions requires a unique activation strategy by the nervous system including altered recruitment order of motor units and decreased motor-evoked potentials [21]. Furthermore, amplitude and area of brain activity is greater during ECC than CON actions, indicating more functional regions of the brain are involved in ECC actions [22]. Given that flywheel RE requires the trainee to accommodate to ECC overload, and acceleration and deceleration, this exercise paradigm would likely prompt substantial adaptations at the cortical level. Although the mechanisms dictating exercise-induced cognitive adaptations are largely unknown, the cortical neuroplasticity reported after RE training in individuals with stroke [23], and the higher activity in specific cortical areas induced by ECC-based RE [22] may play a role in such adaptations.

To this background, this study explored the effects of a 12-week ECC-overload flywheel RE training program of the more-affected lower limb of individuals with chronic stroke on (i) skeletal muscle size, strength and power, (ii) functional performance, and (iii) cognitive function. Given the efficacy of this particular exercise paradigm in more abled populations, we hypothesized there would be substantial muscle hypertrophy and increases in muscle force and power of the more-affected limb. It was also hypothesized these adaptations would be accompanied by improved performance of numerous cognitive functions.

## Methods

### General design

This study compared the effects of unilateral ECC-overload flywheel leg press RE using the more-affected limb (4 sets of 7 repetitions; < 2 min of contractile activity) twice weekly during 12 weeks (training group, TG) with a control group (CG) that followed their daily routines. Before and after the 12-week training period all participants performed maximal dynamic and isometric force and power tests, and m. quadriceps femoris cross sectional area (CSA) and volume were measured using magnetic resonance imaging (MRI). Additionally, cognitive function and functional performance were assessed before and after the training period in both groups.

### Participants

**Fig. 1:**
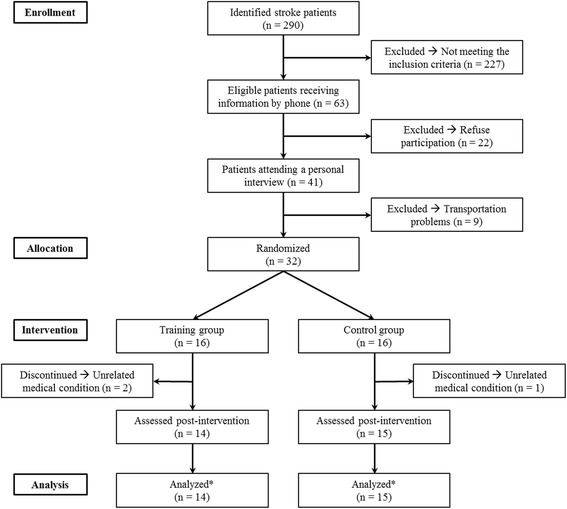
CONSORT flow diagram. * see text for specific number of participants analyzed for each test

Eligible participants were individuals treated from stroke (confirmed by computed tomography or MRI) at Parc Tauli University Hospital (Sabadell, Spain), >40 years of age, ≥6 months post-stroke, mild-moderate hemiparetic gait following stroke (able to walk 20 m with/without assistive device) and ability to perform closed-chain exercise using the prescribed training device. Excluding criteria were unstable angina, congestive heart failure, severe arterial disease, major depression, dementia (<24 on the Mini-Mental State Examination), failure to understand instructions, or chronic pain. Information was provided by phone to 63 eligible patients (Fig. 1). Patients willing to participate (*n* = 32) were randomly assigned (computerized block randomization) to either a training group (TG; *n* = 16) or a control group (CG; *n* = 16). Sample size calculations indicated that with an estimation of 30 % improvement in muscle power [12] and 8 % increase in muscle CSA, 15 participants per group ensured a statistical power of 0.70-0.80. Participants were informed of the purposes and potential risks associated with the interventions before giving their informed written consent to participate. The study protocol was approved by the Ethics Committee at Corporació Sanitaria Parc Tauli (#2013037). This study is registered at ClinicalTrials.gov (#NCT02120846), and was carried out from December 2013 to August 2014. Order, time between tests and time of the day (±2 h) was replicated from pre to post intervention. Prior to any test using the flywheel leg press device participants completed 2 familiarization sessions to define individual machine settings and ensure correct exercise execution.

### Flywheel resistance exercise training

**Fig. 2:**
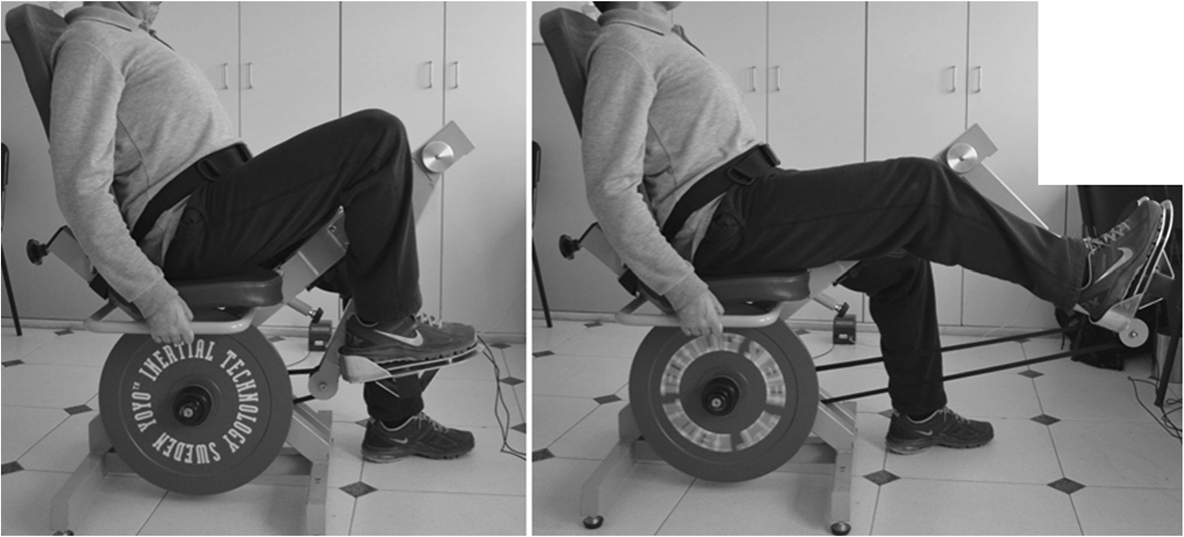
Stroke patient performing resistance exercise on a flywheel leg press device

Participants from TG performed unilateral RE training using the more-affected leg on a flywheel leg press (YoYo™ Technology AB, Stockholm, Sweden) with 0.036 kg*m^2^ moment inertia (Fig. 2), 2 days per week, with >48 h of rest between sessions, for 12 weeks. After a brief standardized warm-up, 4 sets of 7 maximal repetitions were performed following previously validated methodology eliciting ECC overload in individuals with stroke [12]. Briefly, following an initial submaximal repetition to initiate flywheel momentum, seven consecutive maximal repetitions were performed, accelerating rotation of the wheel during CON, and decelerating in the subsequent ECC action. The trainees were requested to push with maximal effort during the entire range of motion in the CON action (from ~70° to almost full extension), where the strap about the flywheel shaft was rolled out. Then, as the strap rewound, they aimed at resisting the inertial force gently during the first third of the ECC action, and then by applying maximal effort to stop the movement at about 70° knee flexion. This strategy allowed for an ECC-overload in force/power values during the last two-thirds of the ECC cycle. Once the flywheel stopped, a subsequent CON action was instantly initiated. A 3-min recovery period was allowed between sets. Peak CON and ECC power was measured in all repetitions (SmartCoach™, Stockholm, Sweden). Real-time performance feedback on peak power was offered to the patients at all times.

### Measurements

#### MRI

**Fig. 3:**
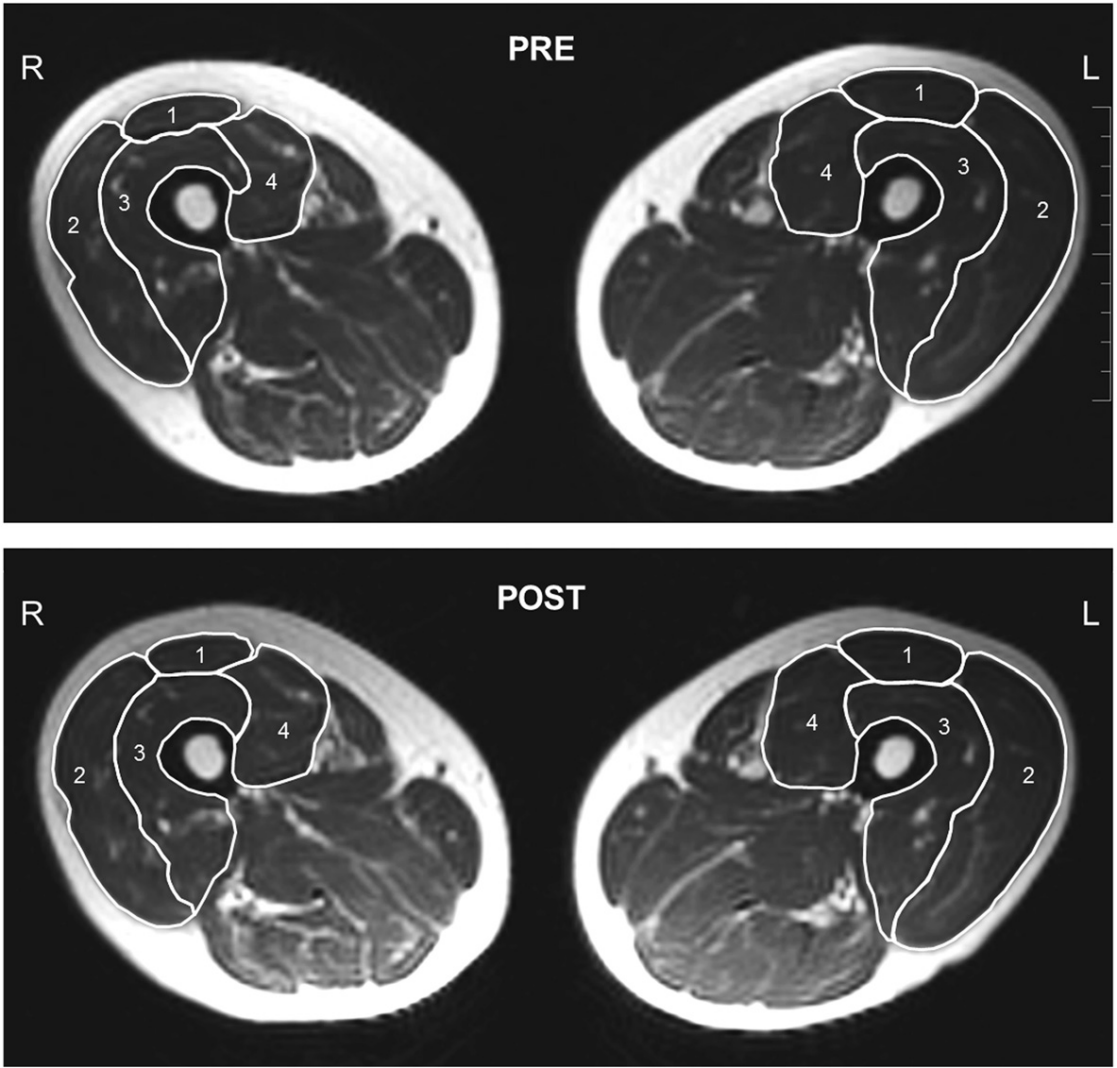
Magnetic resonance images showing thigh muscles (mid-thigh level) of more-affected (R) and less-affected (L) limbs of individual with stroke before (PRE) and after (POST) 12 weeks of ECC-overload flywheel RE training. Quadriceps femoris muscles denoted by numbers are: 1 m. rectus femoris, 2 m. vastus lateralis, 3 m. vastus intermedius, 4 m. vastus medialis. In this particular individual, m. quadriceps femoris volume of more-affected, trained limb increased by 11.8 %

After one hour supine rest, to minimize influence of fluid shift on muscle size [24], cross-sectional images were obtained using a 1.5-Tesla Siemens MR unit (Erlangen, Germany); Turbo spin echo, T_2_ weighted, TE 64 ms, TR 3800 ms, NSA 3, FOV 48 cm, scan time 5 min 2 s, voxel size 1.9x1.9x10 mm. For each participant, 50 continuous images with 10-mm slice thickness were obtained. CSA of each individual quadriceps femoris muscle in order rectus femoris, vastus lateralis, vastus intermedius, and vastus medialis were analyzed in both legs from the first image not displaying m. gluteus maximus and ending with the last image in which rectus femoris appeared. Within this segment, every third image was analyzed by manually encircling the individual muscles (Fig. 3) using RaimViewer software (UDIAT Diagnosis Center, Sabadell, Spain) by research personnel blinded to the origin of any image. The average value of two circumscriptions for each muscle within an image, showing less than 1 % difference was multiplied by slice thickness to obtain muscle volume.

#### Isometric and dynamic force, and peak power

Maximal unilateral isometric force was measured in both legs using a platform with a load cell mounted for either foot on the flywheel leg press at 120° knee angle. The patient was encouraged to push as hard as possible for 5 s against the foot-platform, adjusted and fixed in the desired position using a chain system. Two attempts (3 if the difference between trials was >5 %) were performed. Maximal unilateral dynamic force and peak power tests were performed using the flywheel leg press with 0.036 kg*m^2^ moment inertia during 2 sets of 7 maximal coupled CON-ECC unilateral actions for either limb. Peak dynamic force and power was measured for every coupled CON-ECC repetition using force sensors and encoder systems, respectively.

#### Functional and Dual**-**Task performance

Balance was measured with the Berg Balance Scale [25]. Gait performance was assessed by the Time-Up-and-Go test [26]. Dual-task performance and dual-task cost on walking ability were evaluated using the Talking-While-Walking test [27]. In this test, starting from a standing position, the patient is requested to walk at a fast, still comfortable speed 10 m, turn around and walk back (i.e. single task). Two minutes after, the subject is requested to do the same circuit while simultaneously summing numbers (starting from one, summing three every time; e.g. 1, 4, 7…; i.e. dual task). Dual-task cost on walking was assessed as follows; [(single task time – dual task time)/single task time] x (-100) [28]. The higher the value, the higher the dual-task cost on walking. Spasticity in the more-affected leg was assessed using the Modified Ashworth Scale [29].

#### Cognitive function

Given the lack of data regarding cognitive adaptations in stroke patients after maximal RE, an exploratory approach was employed to analyze the effects of flywheel RE on cognitive functions. Thus, a comprehensive assessment of the most important cognitive functions was performed. Verbal, visual and sustained attention were assessed by the Digits Span Forward subtest from the WAIS-III, the Spatial Span Forward of the Wechsler Memory Scale (WMS-III), and the sustained attention index of the Conners Continuous Performance Test-II (CPT-II). The Rey Auditory Verbal Learning Test evaluated learning and long-term memory. Psychomotor speed was assessed using the index of reaction time from the CPT-II. Processing speed was evaluated using the Stroop Color and Word Test, and the Trail Making Test (TMT) part A. Digits Span Backward subtest of the WAIS-III and Spatial Span Backward subtest of the WMS-III evaluated verbal and visual working memory, respectively. Flexibility, inhibition and verbal fluency, i.e. executive functions, were further evaluated with the TMT part B, third sheet of the Stroop Color and Word Test, the Verbal Fluency Test (FAS) and Semantic Fluency (animals), respectively. All neuropsychological data were collected in raw scores by two neuropsychologists blinded to the intervention. Anxiety and depression (Hospital Anxiety and Depression Scale; only at baseline) and quality of life (SF-36) were also evaluated.

### Statistical analysis

Results are presented as mean (SD), unless otherwise indicated. Baseline descriptive and clinical characteristics were compared between TG and CG using independent Student’s *t*-test (quantitative variables) or Chi-squared test (qualitative variables). Cognitive function, balance, gait and dual-task performance were analyzed by a two-way ANOVA (time x group). Hemispheric lateralization was introduced as a covariate in all models for cognitive variables (ANCOVA) to control for the effect of lateralization across cognitive tasks. Muscle CSA and volume, isometric and dynamic leg press force, and peak power data were examined for TG and CG separately using a two-way ANOVA with factors time and leg. Additionally, a two-way ANOVA (group x time) was used to assess any potential difference between TG and CG. Training data were examined by a one-way ANOVA with repeated measurements over time. Data normality was assessed through histograms and the Shapiro-Wilk test. When significant interactions were found, simple effect tests (t-tests) were employed. The false discovery rate procedure [30] was used to compensate for multiple post hoc comparisons. The level of significance was set at 5 % (*P* < 0.05).

## Results

There were no significant differences (*P* > 0.1) between TG and CG individuals in any descriptive characteristic (Table 1) or measurement at baseline (Table 2 and Table 3). Cognitive and Talking-While-Walking tests were incomplete in subjects who showed expressive aphasia or upper limb spasticity (see Table 3 for details). One TG and one CG patient did not perform the MRI’s due to MRI-incompatible medical prosthesis.

**Table 1:** Baseline characteristics of participants in training group (TG) and control group (CG)

	TG (*n* = 14)	CG (*n* = 15)	Student’s *t*-test/Chi^2^
Age (yr)	61.2 (9.8)	65.7 (12.7)	1.06 (*P* = 0.29)
Height (cm)	165.6 (9.7)	169.3 (9.7)	1.03 (*P* = 0.31)
Weight (kg)	80.4 (15.3)	83.4 (16.0)	0.51 (*P* = 0.61)
Time since stroke (yr)	3.5 (3.6)	4.3 (4.9)	0.96 (*P* = 0.35)
Time under scholarization (yr)	8.9 (3.5)	8.3 (3.6)	0.45 (*P* = 0.66)
Mini-Mental State Examination (a.u)	28.1 (1.8)	27.7 (2.3)	0.53 (*P* = 0.60)
Hospital Anxiety and Depression Scale (Depression)	6.5 (3.7)	5.5 (3.7)	<0.01 (*P* = 0.48)
Hospital Anxiety and Depression Scale (Anxiety)	6.5 (5.1)	7.2 (4.4)	0.69 (*P* = 0.72)
Sex (female/male)	3/11	4/11	0.11 (*P* = 0.74)
Mechanism of stroke (hemorrhagic/ischemic)	5/9	4/11	0.28 (*P* = 0.59)
Localization (cortical/subcortical)	7/7	5/10	0.83 (*P* = 0.36)
Hemispheric lateralization (right/left)	5/9	10/5	4.69 (*P* = 0.09)
Marital status (single/married/divorced)	2/9/2	0/14/1	4.40 (*P* = 0.22)
Dominant side (right/left)	14/0	14/1	0.97 (*P* = 0.33)

Note: To compare baseline values between training group and control group, Student’s *t*-test was used for quantitative variables and Chi-squared test for qualitative variables

**Table 2:** Isometric and dynamic force, peak power, quadriceps femoris (QF) muscles volume and cross sectional area (CSA) in the training group (TG) and the control group (CG) before (PRE) and after (POST) the 12-week eccentric-overload flywheel resistance exercise intervention

		More-affected leg				Less-affected leg			
		PRE	POST	Δ %	95 % CI	PRE	POST	Δ %	95 % CI
Maximal isometric force (N)									
	TG ^c,d^	714.3 (314.4)	856.1 (386.2) ^​#^	19.8	58.03 to 225.47	1128.4 (355.8) *	1148.5 (364.0) *	1.8	-108.05 to 148.23
	CG ^d^	653.4 (327.7)	626.0 (269.7)	-4.2	-108.70 to 53.89	989.1 (363.4) *	983.9 (315.5) *	-0.5	-104.60 to 94.11
Maximal dynamic force (N) ^a^									
	TG ^c^	481.8 (166.3)	548.5 (190.4)	13.8	-1.23 to 134.63	520.5 (192.2)	570.3 (176.2)	9.6	-10.74 to 110.45
	CG ^d^	454.9 (146.9)	431.2 (133.0)	-5.2	-64.07 to 16.71	538.6 (203.3)	545.1 (194.0)	1.2	-37.73 to 50.58
Peak power (W) ^a^									
	TG ^c,d^	89.4 (66.4)	132.5 (87.6) ^#^	48.2	23.25 to 62.92	161.3 (101.7) *	206.7 (117.6) ^#^*	28.1	15.89 to 74.91
	CG ^b,c,d^	92.7 (50.4)	98.1 (55.2)	5.9	-4.48 to 15.37	150.3 (66.8) *	169.6 (66.3) ^#^*	12.9	6.14 to 32.51
QF muscle volume (cm^3^) ^a^									
	TG ^b,c,d^	665.4 (260.0)	727.9 (283.6) ^#^	9.4	37.68 to 87.44	806.3 (263.8) *	810.1 (260.8) *	0.5	-25.53 to 33.12
	CG ^d^	755.6 (258.9)	757.8 (256.9)	0.3	-26.37 to 30.77	861.4 (285.1) *	859.0 (275.3) *	-0.3	-28.47 to 23.59
RF muscle volume (cm^3^)									
	TG ^d^	70.0 (35.4)	70.1 (32.7)	0.1	-5.13 to 5.33	78.6 (34.7)	79.9 (33.8)	1.7	-3.04 to 5.69
	CG	67.9 (29.5)	70.3 (30.4)	3.5	0.17 to 4.58	70.6 (29.3)	72.5 (30.1)	2.7	-1.54 to 5.33
VL muscle volume (cm^3^) ^a^									
	TG ^b,c,d^	230.4 (90.2)	253.5 (96.9) ^#^	10.0	13.71 to 32.54	275.3 (85.3) *	275.6 (84.3)	0.1	-13.16 to 13.91
	CG ^d^	263.9 (93.4)	262.7 (89.6)	-0.5	-14.36 to 11.88	311.2 (118.5) *	308.1 (108.8) *	-1.0	-14.80 to 8.67
VI muscle volume (cm^3^)									
	TG ^b,c,d^	193.2 (79.7)	213.2 (93.6) ^#^	10.3	7.66 to 32.33	240.1 (81.4) *	239.4 (86.8) *	-0.3	-9.84 to 8.53
	CG ^d^	226.7 (90.3)	234.1 (93.9)	3.3	-2.17 to 16.98	255.1 (96.8) *	254.0 (98.1)	-0.4	-9.03 to 6.95
VM muscle volume (cm^3^) ^a^									
	TG ^b,c,d^	171.8 (68.8)	191.2 (71.5) ^#^	11.3	12.30 to 26.39	212.4 (83.0) *	215.2 (76.9) *	1.3	-5.02 to 10.53
	CG ^b,d^	194.4 (70.5)	187.9 (63.9)	-3.3	-16.76 to 3.76	222.5 (68.3) *	224.2 (69.6) *	0.8	-5.90 to 9.38
QF greatest CSA (cm^2^) ^a^									
	TG ^b,c,d^	53.2 (14.2)	57.6 (14.8) ^#^	8.2	2.17 to 6.52	62.6 (13.1) *	63.7 (14.2) *	1.8	-0.83 to 3.06
	CG ^d^	57.5 (16.7)	56.3 (15.9)	-2.0	-3.00 to 0.71	64.8 (15.8) *	64.6 (15.5) *	-0.3	-1.73 to 1.34
QF mean CSA (cm^2^) ^a^									
	TG ^b,c,d^	48.7 (13.1)	53.2 (14.3) ^#^	9.3	3.06 to 6.04	57.7 (13.1) *	58.6 (13.3) *	1.6	-0.67 to 2.47
	CG ^d^	52.6 (14.8)	52.1 (14.3)	-1.0	-2.04 to 1.01	59.4 (14.4) *	59.2 (14.0) *	-0.3	-1.69 to 1.37

Note. 95 % CI; 95 % confidence interval for the Post-Pre difference in absolute values within a group. QF; quadriceps femoris, RF; rectus femoris, VL; vastus lateralis, VI; vastus intermedius, VM; vastus medialis. Significant main effects (*P* < 0.05); ^a^ interaction group x time; ^b^ interaction time x leg; ^c^ main effect of time; ^d^ main effect of leg. Significant simple effects (*P* < 0.05); ^#^vs. pre value within a leg; *vs. more-affected leg for a given time point

**Table 3:** Functional and cognitive variables in training group (TG) and control group (CG) before (PRE) and after (POST) the 12-week eccentric-overload flywheel resistance exercise intervention

	TG				CG			
	n	PRE	POST	Dif (95 % CI)	n	PRE	POST	Dif (95 % CI)
Spasticity (a.u.)	14	1.18 (0.88)	0.94 (0.71)	-0.24 (-0.51 to 0.03)	15	0.79 (0.89)	0.65 (0.71)	-0.13 (-0.40 to 0.14)
Berg Balance Scale (a.u.) ^a, b^	14	42.2 (9.5)	45.9 (9.1) ^#^	3.77 (2.60 to 4.94)	15	45.6 (8.7)	44.0 (9.6) ^#^	-1.64 (-2.77 to -0.51)
Timed-Up-and-Go (s) ^a, b^	14	20.3 (14.5)	18.2 (13.9) ^#^	-2.16 (-3.67 to -0.65)	15	17.6 (16.0)	17.6 (14.8)	0.03 (-1.42 to 1.49)
Single-task circuit time (s) ^b^	14	44.4 (33.4)	40.0 (33.5) ^#^	-4.39 (-7.48 to -1.30)	15	34.7 (30.4)	33.2 (26.0)	-1.45 (-4.42 to 1.53)
Dual-task circuit time (s) ^a^	12	53.5 (47.4)	46.3 (43.5)	-7.19 (-13.07 to -1.31)	14	34.5 (18.5)	36.8 (20.5)	2.30 (-3.58 to 8.19)
Dual-task cost of walking (a.u.) ^a^	12	23.8 (19.7)	17.0 (13.9)	-6.76 (-15.31 to 1.80)	14	27.1 (23.0)	35.4 (31.2)	8.33 (0.15 to 16.53)
Digits Span Forward (a.u.) ^a^	12	7.2 (2.7)	7.4 (2.1)	0.25 (-0.45 to 0.95)	14	7.0 (1.8)	6.7 (2.1)	-0.29 (-0.93 to 0.36)
Digits Span Backward (a.u.) ^a^	12	4.0 (2.2)	4.7 (1.6)	0.67 (-0.10 to 1.44)	14	4.8 (1.5)	4.4 (1.7)	-0.36 (-1.07 to 0.35)
RAVLT Learning (a.u.)	12	35.8 (7.7)	38.3 (8.3)	2.50 (-1.52 to 6.52)	13	37.2 (11.1)	35.8 (11.8)	-1.46 (-5.32 to 2.40)
RAVLT Long-term Recall (a.u.)	12	6.2 (2.7)	5.6 (3.3)	-0.58 (-1.85 to 0.69)	13	6.6 (3.4)	6.5 (3.9)	-0.08 (-1.30 to 1.14)
CPT Reaction Time (a.u.)	14	465.4 (95.6)	457.4 (89.6)	-7.93 (-31.95 to 16.08)	15	447.8 (94.9)	443.8 (82.1)	-4.01 (-27.21 to 19.20)
Spatial Span Forward (a.u.)	14	6.2 (1.8)	6.9 (2.2)	0.64 (-0.24 to 1.53)	15	6.9 (1.6)	6.5 (1.9)	-0.33 (-1.19 to 0.52)
Spatial Span Backward (a.u.)	14	5.4 (2.1)	5.7 (1.8)	0.29 (-0.50 to 1.07)	15	6.1 (2.1)	6.1 (2.2)	-0.07 (-0.83 to 0.70)
Stroop Word (a.u.)	13	74.5 (20.7)	72.1 (22.3)	-2.46 (-14.29 to 9.37)	13	81.2 (22.6)	71.7 (30.5)	-9.46 (-21.29 to 2.37)
Stroop Color (a.u.) ^a^	13	47.2 (18.2)	48.6 (18.0) ^#^	1.46 (-1.85 to 4.78)	13	55.9 (14.8)	54.9 (15.9)	-1.00 (-4.32 to 2.32)
Stroop Word and Color (a.u.)	13	21.6 (12.8)	22.8 (13.9)	1.15 (-2.29 to 4.60)	13	27.5 (12.8)	29.2 (12.2)	1.69 (-1.76 to 5.14)
TMT_A (a.u.)	13	71.6 (29.7)	65.9 (35.1)	-5.77 (-19.15 to 7.61)	15	71.9 (48.4)	67.2 (38.8)	-4.67 (-17.13 to 7.79)
TMT_B (a.u.)	10	206.7 (109.1)	196.6 (129.7)	-10.10 (-42.10 to 21.90)	10	183.2 (165.3)	186.0 (141.9)	2.80 (-29.20 to 34.80)
FAS (a.u.) ^a^	13	21.9 (14.3)	26.6 (15.3) ^#^	4.69 (0.88 to 8.51)	13	25.5 (13.5)	25.4 (15.6)	-0.15 (-3.97 to 3.66)
SF-36 Physical function (a.u.)	13	33.1 (21.8)	38.9 (28.4)	5.77 (-6.16 to 17.70)	15	38.3 (25.6)	44.0 (32.8)	5.67 (-5.44 to 16.77)
SF-36 Physical limitation (a.u.)	13	50.5 (39.6)	65.9 (31.9)	15.39 (-2.73 to 33.50)	15	63.3 (40.5)	62.9 (39.6)	-0.42 (-17.28 to 16.45)
SF-36 Pain (a.u.) ^a^	13	65.2 (36.2)	76.9 (23.1)	11.73 (-6.16 to 29.62)	15	67.3 (39.6)	49.8 (39.1) * ^#^	-17.50 (-34.16 to -0.85)
SF-36 Social function (a.u.)	13	82.7 (19.8)	85.8 (20.1)	3.08 (-10.47 to 16,63)	15	76.7 (29.1)	78.3 (31.5)	1.67 (-10.95 to 14.28)
SF-36 Mental health (a.u.) ^b^	13	61.9 (23.6)	78.1 (15.9)	16.15 (3.02 to 29.29)	15	66.3 (22.6)	71.4 (24.7)	5.02 (-7.22 to 17.24)
SF-36 Emotional problems limitation (a.u.)	13	79.5 (22.7)	89.1 (23.2)	9.62 (-6.71 to 25.94)	15	93.3 (19.9)	87.8 (30.9)	-5.56 (-20.75 to 9.64)
SF-36 Vitality (a.u.)	13	53.9 (20.3)	55.3 (26.9)	1.44 (-14.02 to 16.91)	15	51.0 (23.5)	52.1 (30.9)	1.08 (-13.31 to 15.48)
SF-36 General perception (a.u.)	13	56.5 (17.9)	58.5 (19.9)	1.92 (-7.41 to 11.26)	15	54.0 (22.4)	51.7 (18.9)	-2.33 (-11.02 to 6.36)

Note: n; number of participants, RAVLT; Rey Auditory Verbal Learning Test, CPT; Continuous Performance Test, TMT; Trail Making Test, FAS; Verbal Fluency Test, SF-36; quality of life scale, a.u.; arbitrary units, s; seconds, Dif (95 % CI); Post-Pre difference (95 % confidence interval). Significant main effects (*P* < 0.05); ^a^ interaction group x time; ^b^ main effect of time. Significant simple effects (*P* < 0.05); ^#^vs. pre value within group, *vs TG within a time point

### Flywheel RE training

**Fig. 4:**
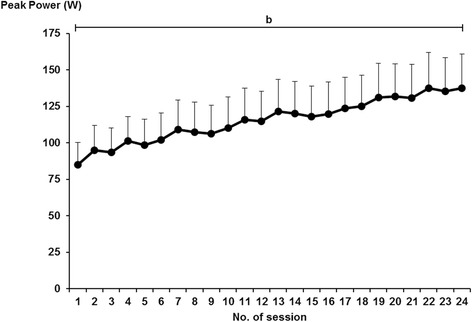
Merged concentric-eccentric leg press peak power (W) of the more-affected limb over 24 training sessions. Significant main effects (*P* < 0.001); b = main effect of session. Data presented as mean ± standard error

All TG patients showed 100 % adherence to the training program. Average peak power rose almost linearly (61 %; main effect of time; *P* < 0.001, F = 14.9) from the first to the last training session (Fig. 4). Time for muscle contractile activity (i.e. time of actual exercise) in any session averaged 1 min and 53 s (±5 s).

### Muscle volume and CSA

TG showed interaction time x leg (*P* < 0.001; F = 22.9) due to increased quadriceps femoris volume in the more-affected (*P* < 0.001), but not the less-affected leg (Table 2). There was an interaction group x time (*P* = 0.04; F = 4.8) due to quadriceps femoris volume gains in the more-affected leg of TG, but not the CG. At large, changes in vastus lateralis, vastus intermedius, and vastus medialis CSA or volume paralleled those of total quadriceps femoris. There was no change for rectus femoris (Table 2). Greatest (*P* = 0.001) and mean (*P* < 0.001) CSA increased only in the more-affected, trained leg of TG (Table 2).

### Isometric and dynamic force, and peak power

Maximal unilateral isometric force (Table 2). There was a main effect of time (*P* = 0.02; F = 6.9), mostly due to increased force of the more-affected leg after the intervention in TG. There was a trend towards an interaction group x time (*P* = 0.06; F = 3.7) mainly due to greater maximal isometric force values in the TG than CG at post.

Maximal dynamic force (Table 2). There was a main effect of time (*P* = 0.03; F = 6.4) due to an increase in dynamic force after the intervention in both the more-affected (13.8 %) and the less-affected (9.6 %) legs in TG. There was an interaction group x time (*P* = 0.03; F = 5.3), due to the improvements for either leg in TG, but not CG.

Merged CON-ECC peak power (Table 2). There was a main effect of time (*P* = 0.01; F = 16.5) due to higher peak power in both legs after training in TG. There was an interaction group x time (*P* = 0.01; F = 7.3) due to overall greater pre-to-post increases in TG, compared with CG.

### Functional performance

Balance (Table 3). There was an interaction group x time (*P* < 0.001; F = 46.8), due to higher Berg Balance Scale scores in the TG (*P* < 0.001; 8.9 %) and slightly lower, yet significant, values in CG (*P* = 0.01; 3.6 %) after the intervention.

Gait performance (Table 3). There was an interaction group x time (*P* = 0.04; F = 4.6). Time to complete the time-up-and-go was reduced in TG after training (*P* = 0.01; 10.6 %). Performance in CG was no different at pre and post.

Dual-task performance (Table 3). There was an interaction group x time (*P* = 0.03; F = 5.6) in the time to complete the circuit during the dual-task trial. While TG employed less time to complete the test (13.4 %), CG increased the time needed to complete the circuit (6.7 %). There was an interaction group x time (*P* = 0.02; F = 7.0) in the dual-task cost on walking. While TG decreased the dual-task cost on walking (28.4 %), values from CG indicated an increase dual-task cost (30.8 %). After compensation for multiple post hoc comparisons, however, none of these within group changes were significant.

Spasticity (Table 3). There were no differences between or within groups.

### Cognitive function

There was an interaction group x time (*P* = 0.02, F = 5.9) in Digits Span Forward (i.e. attention) due to greater values in TG at post compared to pre, while the opposite was found for CG (Table 3). In the Digits Span Backward (i.e. working memory) an interaction group x time was found (*P* = 0.03, F = 5.2). Thus, while TG increased performance in this test, CG showed worse values at post compared to pre (Table 3). There was an interaction group x time (*P* = 0.01, F = 7.7) in Stroop Color test (i.e. speed of information processing) due to greater values in TG (*P* = 0.04) at post compared to pre (Table 3). In addition, an interaction group x time (*P* = 0.02, F = 6.5) was found for FAS (i.e. executive functions), mainly due to increased performance in TG at post compared to pre (*P* = 0.01) (Table 3). Finally, there was an interaction group x time (*P* = 0.02, F = 6.5) in SF-36 pain domain (Table 3). While TG decreased the perception of pain, CG pain perception increased after the intervention period (*P* = 0.03). Thus, perception of pain was higher in CG than in TG at post (*P* = 0.01).

## Discussion

This study assessed the efficacy of 12-week ECC-overload flywheel RE, calling for less than 4 min of contractile activity per week, to induce muscle hypertrophy in individuals with chronic stroke. This novel training paradigm evoked substantial quadriceps femoris hypertrophy of the trained, more-affected lower limb accompanied by marked increases in muscle force and power, and paralleled by improved balance and gait. As the contra-lateral, untrained limb showed augmented dynamic force and power, it follows that neural adaptations should have occurred. Perhaps more intriguing, executive functions, attention and speed of information processing improved after flywheel RE. Given that individuals with stroke not subjected to exercise showed no such effects, we are inclined to attribute the changes noted here to the low volume, high-force muscle contractile activity imposed. Altogether, the results of this pilot randomized controlled trial demonstrate that ECC-overload flywheel RE presents a highly time-efficient method to increase muscle size and function in chronic stroke individuals. It remains to be studied if the improvements in vital cognitive functions noted here, are caused by the unique neural activation strategy typical of ECC-overload flywheel RE.

The current RE paradigm induced a 9.4 % quadriceps femoris hypertrophy of the more-affected limb. Previously, individuals with stroke have shown muscle hypertrophy of this magnitude after 12 weeks of high-volume RE training, comprising more than 4,300 repetitions [31]. The RE program employed here called for less than a sixth (i.e. 672) of that number over 12 weeks. Therefore, flywheel RE emerges as a powerful, time-effective asset to ameliorate muscle mass in individuals with chronic stroke. Attenuating the muscle tissue loss, which occurs secondary to the injury and also from the sedentary life-style consequent to stroke, is fundamental in combatting metabolic and endocrine related disorders jeopardizing health [32].

Participants subjected to high-intensity, ECC-overload flywheel RE, comprising less than 2 min of contractile activity twice weekly, showed 100 % adherence to the current training program. It is noteworthy that the present exercise insult did not affect spasticity, and none of the patients performing the training intervention complained of muscle soreness or fatigue at any time (authors’ observation). Over the 24 scheduled sessions, muscle power output of the more-affected, trained lower limb increased by 61 %, at a weekly rate of ~5 % over the initial 12 weeks (Fig. 4). These results concur with the previous demonstration of a 37 % increase in power of the more-affected leg in a different cohort of individuals with stroke, subjected to 16 sessions of ECC-overload flywheel RE over 8 weeks [12].

Both the more-affected, trained and the less-affected, untrained lower limb of patients from TG showed marked gains in power and dynamic force. This cross-education effect occurs in individuals with stroke after ECC or ECC-overload, but not CON RE [12, 33], underlining the potential role of ECC muscle actions in facilitating central nervous system adaptations. Stroke compromises neural drive to skeletal muscle, and is manifested in neuromuscular dysfunction of both the more- and less-affected limbs [34]. It is plausible that the unique neural command required to execute ECC muscle actions [21] provides a powerful stimulus to counteract this effect.

Cognitive impairments and interferences between functional and cognitive tasks affect activities of daily living after stroke [13, 14, 35]. Hence, the improvements reported in dual-task performance after ECC-overload flywheel RE should be regarded as a promising outcome of the current training paradigm. Furthermore, executive functions were enhanced in response to 12 weeks of ECC-overload flywheel RE, together with improved attention and speed of information processing. The benefits of physical exercise on cognitive function have been investigated in different populations [18, 36], including individuals with stroke [37–41]. Similar to the current findings, exercise-induced adaptations of cognitive functions typically include improved executive functions. However, the vast majority of studies have employed high-volume aerobic or combinations of exercise modes, in settings where social interaction likely impacts cognitive outcomes [39, 42]. Our data suggest that individually and strictly controlled supervised sessions of maximal-intensity ECC-overload RE, comprising 4 min of weekly contractile activity, could improve cognition, and in particular, executive functions in individuals with stroke. This would suggest that the exercise stimuli *per se* rather than the social environment, was responsible for these changes. Albeit the mechanisms behind such exercise-induced adaptations are currently unknown [43], the RE modality employed here offers a stimulus that is unique [20–22, 44] and likely amplifies such adaptations. A potential explanation for the current findings is that ECC muscle actions facilitate activity of specific brain regions, including the cingulate cortex [45], an area involved in the cerebral circuits controlling working memory [46, 47], verbal fluency [48] and pain perception [49], variables that improved after the current flywheel RE protocol. Future studies should employ functional MRI or electroencephalography to study potential central nervous system adaptations after flywheel RE training.

The gains in muscle size, power and force showed here, translated into enhanced functional capacities, with markedly improved balance and short-distance gait performance after the intervention. Indeed, significant clinical gains [50] were evident in the 2-s faster time-up-and-go test performance. However, augmented Berg Balance Scale scores did not attain the minimal clinically significant difference proposed for the elderly, i.e. 8 points [51]. Yet, while patients possessed increased risk of fall, as indicated by the <45 Berg Balance Scale score [52] at baseline, balance was improved after 12 weeks training. Taken together, ECC-overload flywheel RE induces major muscle hypertrophy accompanied by increased power and force, and improved functional performance (i.e. short-distance gait and balance) adding to our previous findings [12].

This well-controlled pilot randomized clinical trial is not without limitations. Larger multicenter trials employing active controls performing conventional physiotherapy, occupational therapy or other types of RE training, and comprising long-term follow-up are warranted to manifest our novel findings and explore the mechanisms behind ECC-overload RE-induced cognitive adaptations. In addition, the hemispheric lateralization of stroke should be controlled for in the randomization process. The current study design dealt with this potential flaw by introducing hemispheric lateralization as a covariate in all statistical models for cognitive variables. If meeting the premises of the present outcome, we suggest the ECC-overload flywheel RE paradigm should be implemented in rehabilitation settings as a safe and viable method to promote neuromuscular and cognitive adaptations, while significantly reducing time needed for therapy.

## Conclusions

Short-intense bouts of ECC-overload flywheel RE over 12 weeks induced unprecedented muscle hypertrophy of the more-affected, trained lower limb paralleled by marked increases in force and power of either limb, in individuals with chronic stroke. These changes were accompanied by enhanced balance and gait, without affecting muscle spasticity. Not less important, this intervention, calling for less than 2 min of muscle contractile activity per session, improved executive functions, attention and speed of information processing. Collectively, the results merit this particular flywheel RE paradigm as a highly time-effective rehabilitation tool to aid individuals with chronic stroke in boosting muscle size and function, functional performance, and cognitive functions.

## Abbreviations

CG: control group
CON: concentric muscle action
CPT-II: Conners continuous performance test-II
CSA: cross sectional area
ECC: eccentric muscle action
FAS: verbal fluency test
MRI: magnetic resonance imaging
RE: resistance exercise
SF-36: short form 36 health survey
TG: training group
TMT: trail making test
WMS-III: Wechsler Memory Scale
